# Modulating Expression of Endogenous Interleukin 1 Beta in the Acute Phase of the Pilocarpine Model of Epilepsy May Change Animal Survival

**DOI:** 10.1007/s10571-022-01190-y

**Published:** 2022-01-21

**Authors:** V. D. B. Pascoal, R. B. Marchesini, M. C. P. Athié, A. H. B. Matos, F. F. Conte, T. C. Pereira, R. Secolin, R. Gilioli, J. M. Malheiros, R. S. Polli, A. Tannús, L. Covolan, L. B. Pascoal, A. S. Vieira, E. A. Cavalheiro, F. Cendes, I. Lopes-Cendes

**Affiliations:** 1 Department of Translational Medicine, School of Medical Sciences, University of Campinas (UNICAMP), and the Brazilian Institute of Neuroscience and Neurotechnology (BRAINN), Tessália Vieira de Camargo, 126, Cidade Universitária “Zeferino Vaz”, Campinas, SP 13083-887 Brazil; 2 Department of Basic Science, Fluminense Federal University, Nova Friburgo, RJ Brazil; 3 Department of Biology, Faculty of Philosophy, Sciences and Letters at Ribeirao Preto, University of Sao Paulo (USP), Ribeirao Preto, SP Brazil; 4 Multidisciplinary Centre for Biological Investigation (CEMIB), University of Campinas (UNICAMP), Campinas, SP Brazil; 5 Centro de Imagens e Espectroscopia por Ressonancia Magnetica (CIERMag), Institute of Physics, University of Sao Paulo (USP), Sao Carlos, SP Brazil; 6 Department of Physiology, Federal University of Sao Paulo (UNIFESP), Sao Paulo, SP Brazil; 7 Institute of Science and Technology, Federal University of São Paulo, São José dos Campos, SP Brazil; 8 Laboratory of Cell Signaling, School of Medical Sciences, University of Campinas – (UNICAMP), Campinas, SP Brazil; 9 Department of Structural and Functional Biology, Institute of Biology, University of Campinas – (UNICAMP), Campinas, SP Brazil; 10 Department of Neurology and Neurosurgery, Federal University of Sao Paulo, (UNIFESP), Sao Paulo, SP Brazil; 11 Department of Neurology, School of Medical Sciences, University of Campinas – (UNICAMP); and the Brazilian Institute of Neuroscience and Neurotechnology (BRAINN), Campinas, SP Brazil

**Keywords:** Mesial temporal lobe epilepsy, Neuroinflammation, RNA interference in vivo, Animal model

## Abstract

The pilocarpine-induced (PILO) model has helped elucidate the electrophysiological and molecular aspects related to mesial temporal lobe epilepsy. It has been suggested that the extensive cell death and edema observed in the brains of these animals could be induced by increased inflammatory responses, such as the rapid release of the inflammatory cytokine interleukin 1 beta (Il1b). In this study, we investigate the role of endogenous Il1b in the acute phase of the PILO model. Our aim is twofold. First, we want to determine whether it is feasible to silence Il1b in the central nervous system using a non-invasive procedure. Second, we aim to investigate the effect of silencing endogenous Il1b and its antagonist, Il1rn.We used RNA interference applied non-invasively to knockdown *Il1b* and its endogenous antagonist *Il1rn.* We found that knocking down *Il1b* prior to pilocarpine injection increased the mortality rate of treated animals. Furthermore, we observed that, when exposing the animals to more Il1b by silencing its endogenous antagonist *Il1rn*, there was a better response to *status epilepticus* with decreased animal mortality in the acute phase of the PILO model. Thus, we show the feasibility of using a novel, less invasive approach to study genes involved in the inflammatory response in the central nervous system. Furthermore, our results provide suggestive evidence that modulating endogenous Il1b improves animal survival in the acute phase of the PILO model and may have effects that extend into the chronic phase.

**Supplementary Information:**

The online version contains supplementary material available at 10.1007/s10571-022-01190-y.

## Introduction

It has been estimated that nearly half of adult patients with epilepsy have mesial temporal lobe epilepsy (MTLE). Among these, only about 60% achieve optimal seizure control with clinical treatment, making MTLE one of the most frequent and severe forms of epilepsy (Sander 1993; Mattson 1994; Borges et al. 2004). One approach used to investigate the pathophysiology of MTLE is the study of experimental animal models, which present epileptogenicity similar to that observed in so-called “epileptic” human tissue studied ex vivo. Among the various models available, the pilocarpine-induced (PILO) model is well-established and presents extensive molecular, histological, physiological, and phenomenological characterization (Cavalheiro et al. 1991; Smolders et al. 1997; Hamani et al. 1999; Raza et al. 2001; Garzillo and Mello 2002; Bender et al. 2004; Dudek and Sutula 2007; Curia et al. 2008; Ravizza et al. 2008; Carter et al. 2011; van Eijsden et al. 2011; Mazzuferi et al. 2012; Scharfman and Pierce 2012; Pearson et al. 2014; Gonzalez et al. 2015; Cardim et al. 2016). In addition, the sequence of events observed in the different phases of the PILO model is somewhat similar to that seen in some patients with MTLE in whom an initial precipitating event can be identified (Bender et al. 2004). Furthermore, the histological characteristics of the hippocampal lesion observed in the chronic phase of PILO animals are comparable to that found in patients with MTLE and typical findings of mesial temporal sclerosis (Dudek and Sutula 2007; Curia et al. 2008). However, the lesion in the PILO model tends to be more extensive, with spread to extra-hippocampal regions (Fabene et al. 2003; Nairismagi et al. 2004).

It has been well characterized that one important biological phenomenon observed in the acute phase of the PILO model is cell death, which is believed to be caused by apoptosis (Voutsinos-Porche et al. 2004) and necrosis (Malheiros et al. 2014), as well as cell swelling due to massive edema (Ong et al. 2009). Furthermore, it has been suggested that these events could be induced by an inflammatory response (Bernardino et al. 2005; Ravizza et al. 2008). Therefore, changes in the expression of genes related to inflammatory pathways such as interleukins, interferons, tumor necrosis factor (*Tnf*), and others have been studied in the acute phase of the PILO model (John et al. 2005; Benson et al. 2015; Li et al. 2017). Because of the increased expression of interleukin 1 beta (*Il1b*) in the acute phase of the PILO model (Voutsinos-Porche et al. 2004), this cytokine has emerged as a candidate key player in the molecular mechanisms underlying the damage induced by pilocarpine. Although a large body of literature has been produced regarding the investigation of Il1b in the PILO model (for a review, see Rijkers et al. 2009), there are still many unanswered questions, and the matter is far from resolved (Arzimanoglou et al. 2002). In this study, we investigate the role of endogenous Il1b in the acute phase of the PILO model. Our aim is twofold. First, we want to determine whether it is feasible to silence Il1b in the central nervous system using a non-invasive procedure. Second, we aim to investigate the effect of silencing endogenous Il1b and its antagonist, Il1rn. To accomplish these goals, we used a non-invasive and specific tool to modify gene expression—RNA interference (RNAi)—and delivered specially constructed small interfering RNA molecules (siRNA) to inhibit endogenous *Il1b* and *Il1rn* in the central nervous system (CNS).

## Methods

### Animal Handling

We used specific-pathogen-free (SPF) 8-week-old male rats (HanUnib:WH) obtained from the Multidisciplinary Centre for Biological Investigation (CEMIB) at the University of Campinas (UNICAMP). The experimental protocols were approved by the Ethics Committee in Animal Experimentation at UNICAMP and were performed in a biosafety level 2 animal facility, following national and international recommendations for the ethical and safe handling of animals. Rats were divided into four groups (five animals per group) as follows: (i) one control group injected with phosphate-buffered saline (PBS), (ii) a second control group injected with an irrelevant siRNA directed against green fluorescent protein (GFP) (siGFP), (iii) an experimental group injected with siRNA directed against *Il1b* (siIl1b), and (iv) an experimental group injected with siRNA directed against *Il1rn* (siIl1rn). Animals were kept during the experimental period in micro-insulators with sterilized wood shavings in a ventilated rack unit equipped with a HEPA filtration system and with 12-h light/12-h dark photoperiod. Nuvital CR-1 pelleted food and water sterilized by autoclaving were provided ad libitum.

Animals treated with pilocarpine were euthanized at different times for the different experiments, because we analyzed molecular changes occurring in the silent phase 5 days after *status epilepticus* (SE), and the histological changes occurring in the chronic phase, 90 days after SE.

### Peptides and siRNA

The rabies virus glycoprotein (RVG)-9R peptide (YTIWMPENPRPGTPCDIFTNSRGKRASNGGGGRRRRRRRRR) was synthesized and purified by using high-performance liquid chromatography at the Bio-Synthesis Inc. (Lewisville, TX, USA); the nine C-terminal arginine residues were D-arginine. siRNA were designed against the genes *Il1b* and *Il1rn* using the *Strand Analysis* program (Pereira et al. 2007). Subsequently, the sequence was checked in the Blast program to confirm that it did not have homology for any other gene expressed in the rat. The siRNA were purchased from IDT (Coralville, IA, USA).

The siRNA sequences are:

siIl1b sense: 5′ UGACCCAUGUGAGCUGAAAGC 3′;

siIl1b antisense: 5′ UUUCAGCUCACAUGGGUCAGA 3′;

siIl1rn sense: 5′ AGGCACUCCCAGAGUAUGUGU 3′;

siIl1rn antisense: 5′ ACAUACUCUGGGAGUGCCUUC 3′;

siGFP sense: 5′ CAGGCUACUUGGAGUGUAUUU 3′;

siGFP antisense: 5′ AUACACUCCAAGUAGCCUGUU 3′.

### Delivery of siRNA::Peptide Complexes

siRNA::peptide complexes (at a molar ratio of 1:10 siRNA to peptide) were delivered through the intravenous (i.v.) route into the lateral tail vein; complexes were prepared in 500 μL of PBS containing 5% glucose. To identify the best experimental conditions (mass of siRNA and number of injections), animals were injected with different amounts of 25–50 μg of siRNA, with one or two injections per animal (with an 8-h interval between injections). Brains were harvested 48 h after the first injection. To characterize the *Il1b* silencing time course, rats were given two injections of 25 μg of siRNA (with an 8-h interval between injections), and brains were collected at the following time points: 24, 48, 72, 96, and 120 h after the first injection of siRNA. To assess the silencing effect in different anatomical areas, the brain was divided into five regions as follows: the hippocampus, the frontal cortex, the olfactory bulb, the cerebellum, and the brain stem. The spinal cord was also analyzed (Pascoal 2010).

### Real-Time Polymerase Chain Reaction (qPCR)

To quantify the transcripts of interest, we used three animals per group for each time point. Total RNA was isolated from the brain using the TRIzol reagent (Invitrogen, Carlsbad, CA, USA). RNA was reverse transcribed with SuperScript III and random primers (both from Invitrogen) according to the manufacturer’s protocol. qPCR was performed with 40 ng of complementary DNA using the TaqMan System (Applied Biosystems 7500 Real-Time PCR, Foster City, CA, USA). The amplification conditions were as follows: 50 °C for 2 min, 95 °C for 10 min followed by 40 cycles of 95 °C for 15 s (melting step) and 60 °C for 1 min (annealing/extension step).

To assess siRNA specificity, the expression profile of two biologically related genes (*Ptgs2* and *Nfkb*) and two unrelated genes (*Plat* and *Ntrk2*) were determined by using qPCR in all experimental groups. We used *Gapdh* and *Hprt1* internal controls for normalization because previous analyses revealed they are the steadiest endogenous controls in the regions analyzed (Tanic et al. 2007). The relative gene expression was calculated by the comparative threshold cycle method (Livak and Schmittgen 2001). All primers and probes were obtained from Applied Biosystems.

### Assessment of Blood–Brain Barrier (BBB) Integrity by Magnetic Resonance Imaging

To determine whether siRNA::RVG-9R promotes BBB lesions, magnetic resonance imaging (MRI) was performed using gadolinium diethylenetriaminepentacetate (Gd-DTPA) as a paramagnetic contrast. Diffusion of the contrast observed after siRNA::peptide injection would indicate evidence of damage to the BBB (Prior et al. 2004). MRI was performed at the CIERMag on a Bruker electronic (Avance III under Paravision 5) adapted to a 2-Tesla field 31 cm horizontal superconducting magnet (Oxford Instruments 85310HR, Abingdon, UK). A crossed saddle radiofrequency coil (Papoti et al. 2010) was used as a head probe for the animals. For all animals, the image acquisition started 15 min after injection of 0.5 M Gd-DTPA (1 mL/kg, i.v.) injection. After deep anesthesia, induced by a mixture of ketamine [120 mg/kg, intraperitoneal (i.p.)] and xylazine (9 mg/kg, i.p.), T1-weighted images were collected using a fast low angle shot (FLASH) sequence. The parameters used were: time of echo (TE) 5.0 ms, repetition time (TR) 200 ms, flip angle 90°, 192 × 192 points over a field of view of 4 × 4 cm^2^, slice thickness 1 mm, interslice gap 1.3 mm, and 16 coronal slices covering almost the entire brain.

### Pilocarpine Treatment

Rats were injected with methylscopolamine (1 mg/kg in saline, i.p.; Sigma-Aldrich, St. Louis, MO, USA) 30 min before injecting pilocarpine hydrochloride (300 mg/kg in saline, i.p.; Merck, Quimica, Brazil) to prevent peripheral cholinergic side effects. Immediately after pilocarpine injection, animals were placed in a cage with paper-covered sawdust to avoid choking on sawdust during *status epilepticus* (SE; *status* was defined as a condition of continuous seizures lasting longer than 30 min). About 40 min after the pilocarpine injection, approximately 70% of rats had developed SE consisting of generalized motor seizures, which were interrupted 120 min after their onset by diazepam administration (10 mg/kg, i.p.) to reduce the mortality rate. Only rats with seizures that achieved class five motor behavior as described in the Racine Scale as loss of postural tone, jumping, and generalized tonic–clonic activity, and entered SE after pilocarpine injection, were used for subsequent analyses because previous experiments showed that these rats develop spontaneous recurrent seizures in the chronic phase (Turski et al. 1984; Arida et al. 1999).

### Brain Excision and Tissue Preparation for Nissl Staining

Animals were euthanized 90 days after pilocarpine-induced SE (chronic phase). For this analysis, three animals per group were deeply anesthetized with a mixture of ketamine hydrochloride (120 mg/mL, i.p.) and xylazine (9 mg/mL, i.p.) and then rapidly perfused through the heart with four solutions in the following order: (i) 25 mL of Millonig’s buffer; (ii) 50 mL of sodium sulfide fixative 0.1% in Millonig’s buffer; (iii) 100 mL of glutaraldehyde 3%; (iv) 200 mL of sodium sulfide fixative 0.1% in Millonig’s buffer. Millonig’s buffer was prepared 1 day before perfusion using 3.88 g of sodium hydroxide, 16.56 g of sodium phosphate monobasic, and 0.02 g of calcium chloride per liter of distilled water. The brains were cut on a cryostat (32-µm-thick sections); all sections corresponding to an interval between − 2.8 and − 5.8 mm from the bregma (Paxinos and Watson 2013) were collected, comprising a total sectioned extent of 3 mm per animal. Every third consecutive section was mounted onto a gelatin-coated slide and then processed for Nissl staining to assess the distribution and severity of neuronal damage, which was determined by cells/µm^2^. The number of cells was counted only in the dorsal hippocampal subregions (dentate gyrus, CA1, CA3, and hilus), bilaterally, and in triplicate. This count was conducted by a researcher unaware of the treatment of the experimental groups with the help of an optical microscope (Nikon, Eclipse E600FN) with a checkered grid. The area of interest had two forbidden lines (exclusion) and two acceptable lines (inclusion). The cells that touched the forbidden lines were not counted, but those that touched the acceptable lines and were within the area of interest were counted. Each hippocampal subregion was divided into three fields, each field with 5000 µm^2^. Thus, the outcome was determined by the average of field values.

### Statistical Analysis

The Kruskal–Wallis test was used to assess differences between distinct groups. The Mann–Whitney test was used to evaluate differences between brain regions and gene expression changes within the same group. Gene silencing was confirmed in comparison with controls. For qPCR, analysis of variance (ANOVA) followed by Tukey’s post hoc test was used to analyze messenger RNA (mRNA) levels; *p* < 0.05 was considered a significant difference. For phenotype analysis, including mortality rate, we used one-way ANOVA followed by Tukey’s post hoc test (seizure analysis). Differences in the number of cells counted in the hippocampal subregions (average field values) were accessed by one-way ANOVA followed by Tukey’s post hoc test. Statistical analyses were carried out with the BioEstat v.5.0 program.

## Results

### Two Tail Injections of Il1b siRNA::RVG-9R Successfully Decreased Il1b Brain Expression with no Off-Target Effects

**Fig. 1:**
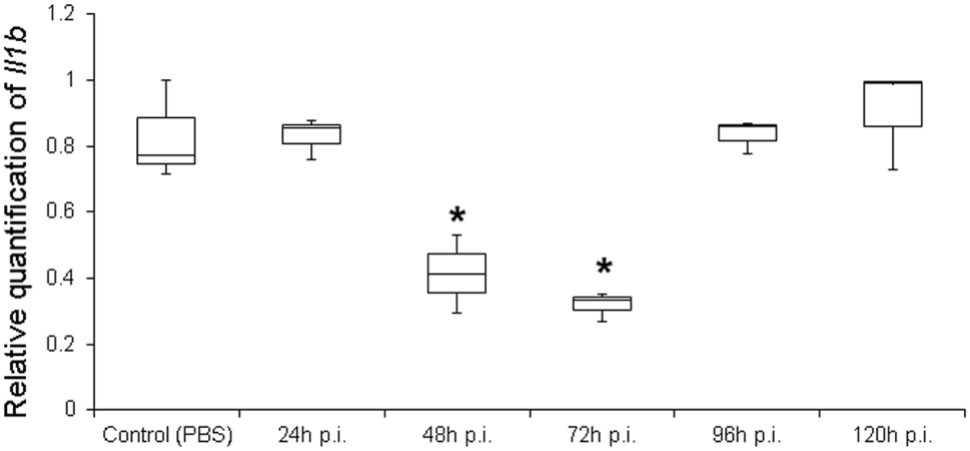
Gene silencing effect of *siIl1b* over time. Animals were injected twice (8-h interval between injections) with 25 μg of *siIl1b* in the tail vein. Gene expression was quantified in the whole brain by real-time polymerase chain reaction (qPCR). Control animals were injected with phosphate-buffered saline (PBS) or siGFP (data not shown). All groups were composed of three animals. Asterisk indicates significant gene silencing (Kruskal–Wallis test, *p* < 0.05). p.i., post-injection

We introduced a few modifications to the original procedure developed in mice (Kumar et al. 2007). First, we tested the possibility of reducing the number of injections from three to two (with an 8-h interval between injections). We observed that gene silencing using siRNA against *Il1b* (siIl1b) was dose-dependent, as the strongest knockdown effects (> 80% reduction in mRNA in the whole rat brain 48 h post-injection) was achieved using two tail injections of 50 μg of siRNA (Supporting information, Fig. S1). However, because two injections of 25 μg of siRNA yielded satisfactory gene silencing results (*Il1b* mRNA reduction close to 60%), all subsequent experiments were performed with this smaller mass to avoid possible off-target effects, as recommended in the literature (Tschuch et al. 2008; Caffrey et al. 2011). Therefore, using only two intravenous tail injections, we observed that gene expression started to decline after 24 h; the effect lasted for more than 3 days (Fig. 1).

Transvascular tail injections of siIl1b resulted in significant gene silencing in different areas of the CNS (Supporting information, Fig. S2), which was observed 24 h post-injection (Fig. 1), with the maximum effect on mRNA levels occurring at 72 h after siIl1b injection, as determined by qPCR. Normal mRNA levels were restored by 96 h after siIl1b injections (Fig. 1).

**Fig. 2:**
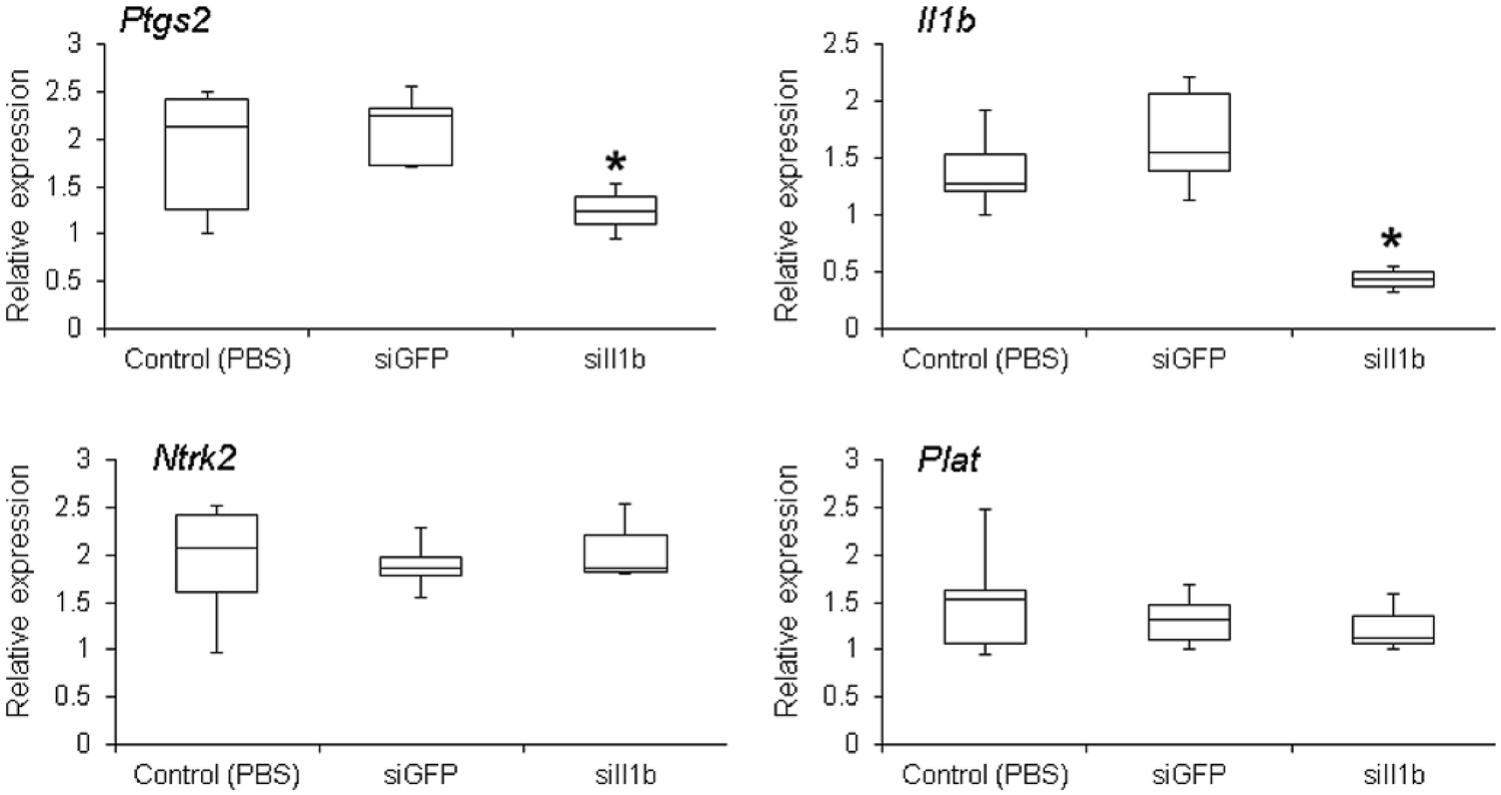
Assessment of RNA interference specificity. Relative gene expression quantification [real-time polymerase chain reaction (qPCR)] of *Ptgs2*, *Il1b*, *Ntrk2*, and *Plat* in animals that received two injections of 25 μg of siRNA (8-h interval between injections). Brains were collected and RNA was extracted 72 h after the first injection. As expected, phosphate-buffered saline (PBS) alone or siGFP did not affect the expression of any of the assessed genes. By contrast, siIl1b led to the silencing of both *Il1b* and *Ptgs2* (which are in the same biological pathway as *Il1b*), whereas *Ntrk2* and *Plat* (which are not biologically related to *Il1b*) were not affected. Each experiment was performed in the whole brain and extracted from five animals. Asterisk indicates significant gene silencing (Kruskal–Wallis test, *p* < 0.05)

Two different strategies were used to assess RNAi specificity. Initially, we tested the effects of siIl1b on the expression levels of *Ptgs2* and *Nfkb* (Supporting information, Fig. S1). As expected, two injections of 25 μg led to an evident knockdown of both genes, an expected outcome because both genes are induced by Il1b (Soloff et al. 2006). Surprisingly, increasing the mass of siIl1b to 50 μg did not reduce *Ptgs2*. Indeed, we observed an increase in the expression of *Ptgs2*, making its expression similar to controls. This effect could be an indication of an off-target effect as *Ptgs2* could be induced by other biological pathways (Kaidi et al. 2006; Manieri et al. 2012). By contrast, siGFP (used as negative control) did not affect the expression of either of these genes (Supporting information, Fig. S1), clearly showing that RNAi was dependent on target sequence complementarity. The second approach to check for RNAi specificity focused on the expression of two genes not related to *Il1b*: *Ntrk2* and *Plat*. In this experiment, using siIl1b and siGFP, there were no gene knockdown effects, as expected (Fig. 2).

### siRNA::RVG-9R Complexes did not Compromise BBB Integrity

**Fig. 3:**
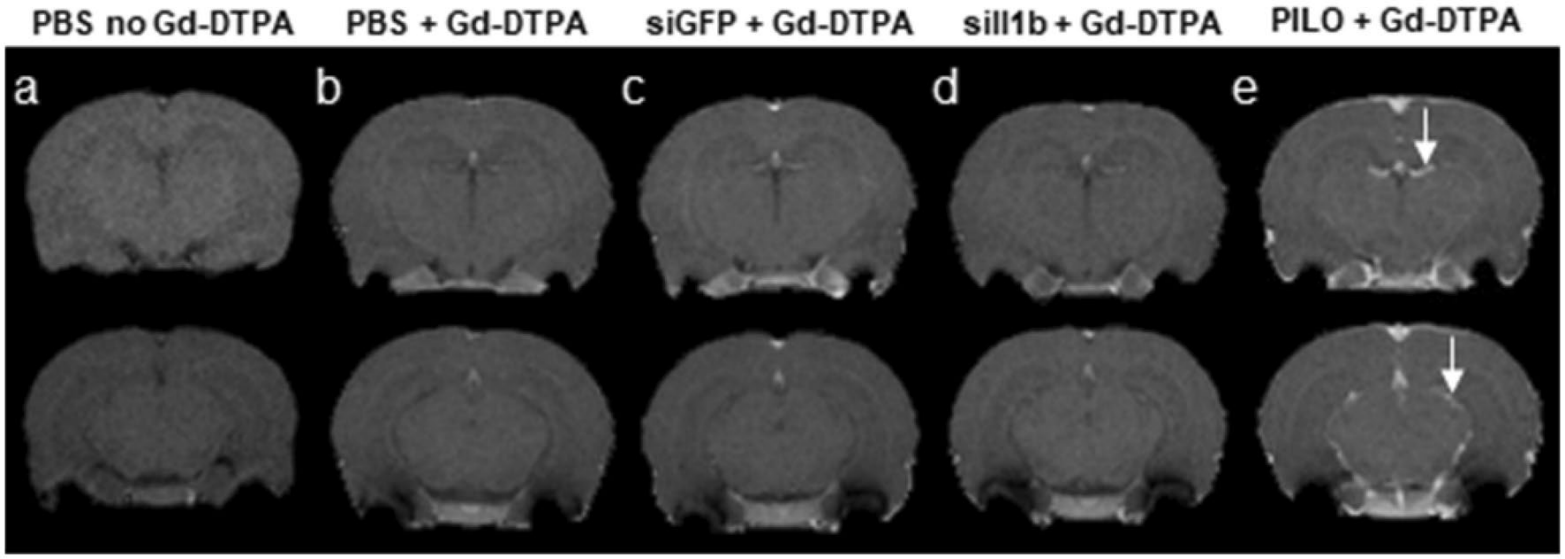
Representative magnetic resonance images of animals used in our experiments with RNA interference. We selected two slices near the middle of the cerebrum and another one in the hippocampal region: **a** images from an animal that was injected with PBS but not paramagnetic contrast (gadolinium diethylenetriaminepentacetate [Gd-DTPA]); **b** animals injected with phosphate-buffered saline (PBS, control) followed by Gd-DTPA; **c** animals injected with siGFP::RVG9R followed by Gd-DTPA; **d** animals injected with siIl1b::RVG9R followed by Gd-DTPA; and **e** animals injected with Gd-DTPA 2 h after *status epilepticus* induced by pilocarpine (PILO) injection (positive control). All groups were composed of three animals. The white arrows indicate the presence of Gd-DTPA (intensified signal) within the brain as a marker of BBB damage

To verify whether the crossing of siRNA::RVG-9R complexes from the periphery to the CNS could induce significant damage to the BBB, we performed MRI in animals from two RNAi control groups (injected with PBS or siGFP) and in one RNAi experimental group (injected with siIl1b) using Gd-DTPA as a paramagnetic contrast. As a positive control, we used animals that remained in pilocarpine-induced SE for 2 h, which is known to damage the BBB (Marchi et al. 2007). After image acquisition, we selected two slices near the middle of the cerebrum and one in the hippocampal region for visual analysis (Fig. 3). There was no sign of BBB damage, although it is noteworthy that this approach considers the limitation of the paramagnetic contrast used (Gd-DTPA, molecular weight nearly 550 g/mol), which can only assess BBB integrity for molecules of an equivalent size or larger. These data were enough to confirm that the complex of siRNA::RVG9R did not cause significant damage to the BBB.

### Il1b mRNA Expression Showed a Rapid Increase 1 h After SE in the PILO Model

**Fig. 4:**
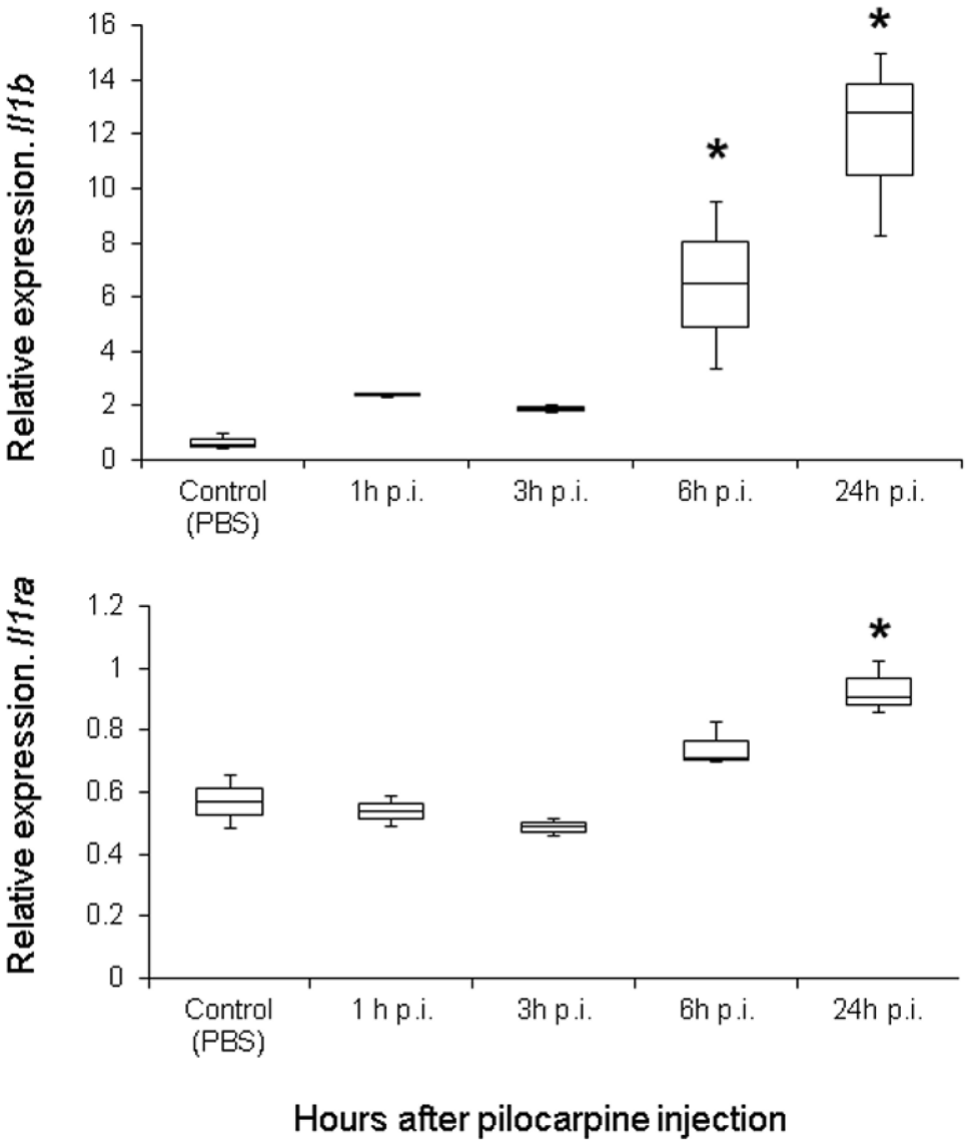
*Il1b* and *Il1rn* gene expression profiles over time in the brain after pilocarpine injection. The expression profiles were generated by using real-time polymerase chain reaction (qPCR) of both genes 1, 3, 6, and 24 h post-injection (p.i.) of pilocarpine and compared with control (no siIl1b or pilocarpine administration). We observed an increase in *Il1b* expression at 6 h p.i. All groups were composed of three animals. Asterisk indicates significant changes in gene expression (Kruskal–Wallis test, *p* < 0.05)

To achieve timely knockdown of *Il1b* in the brain, it was important to determine more precisely the time frame when the increase in expression takes place; therefore, we quantified *Il1b* transcripts at short intervals (1, 3, 6 and 24 h) after SE. Surprisingly, we observed a very early and rapid increase in *Il1b* mRNA starting in the first hour after SE (Marchesini 2011). Continuing observations showed that within 6 h after SE, *Il1b* mRNA expression showed a ten-fold increase compared with control animals (Fig. 4). In addition, we observed a more modest upregulation of *Il1rn* (Fig. 4). Considering the very early increase in *Il1b* expression in the course of pilocarpine-induced SE, we decided to inject siIl1b 48 h before intraperitoneal pilocarpine administration. By doing so, we expected that, at the observed time of increased *Il1b* expression, injection of siIl1b would be able to control the striking pilocarpine-mediated *Il1b* upregulation.

### Il1b Knockdown Led to a More Severe Phenotype and Increased Animal Mortality in the Acute Phase of the PILO Model

For the animal mortality analysis, we injected 50 animals with pilocarpine; 42 (84%) developed SE. However, three animals were excluded from further experiments due to technique failure during siRNA injection in the tail vein. Thus, we had a total of 39 animals for the animal mortality analysis.

There were no significant differences in the death rates among the groups in the period immediately following SE (first day): controls (8/19) = 19%; animals injected with siIl1b (4/9) = 22%; and animals injected with siIl1rn (2/11) = 25%. Nonetheless, 5 days after SE, we observed significant differences in animal mortality, as follows: controls (9/19) = 47%; animals injected with siIl1b (6/9) = 66%; and animals injected with siIl1rn (3/11) = 27% (*χ*^2^, *p* < 0.05 compared with the control group) (Supporting information, Table S1).

**Fig. 5:**
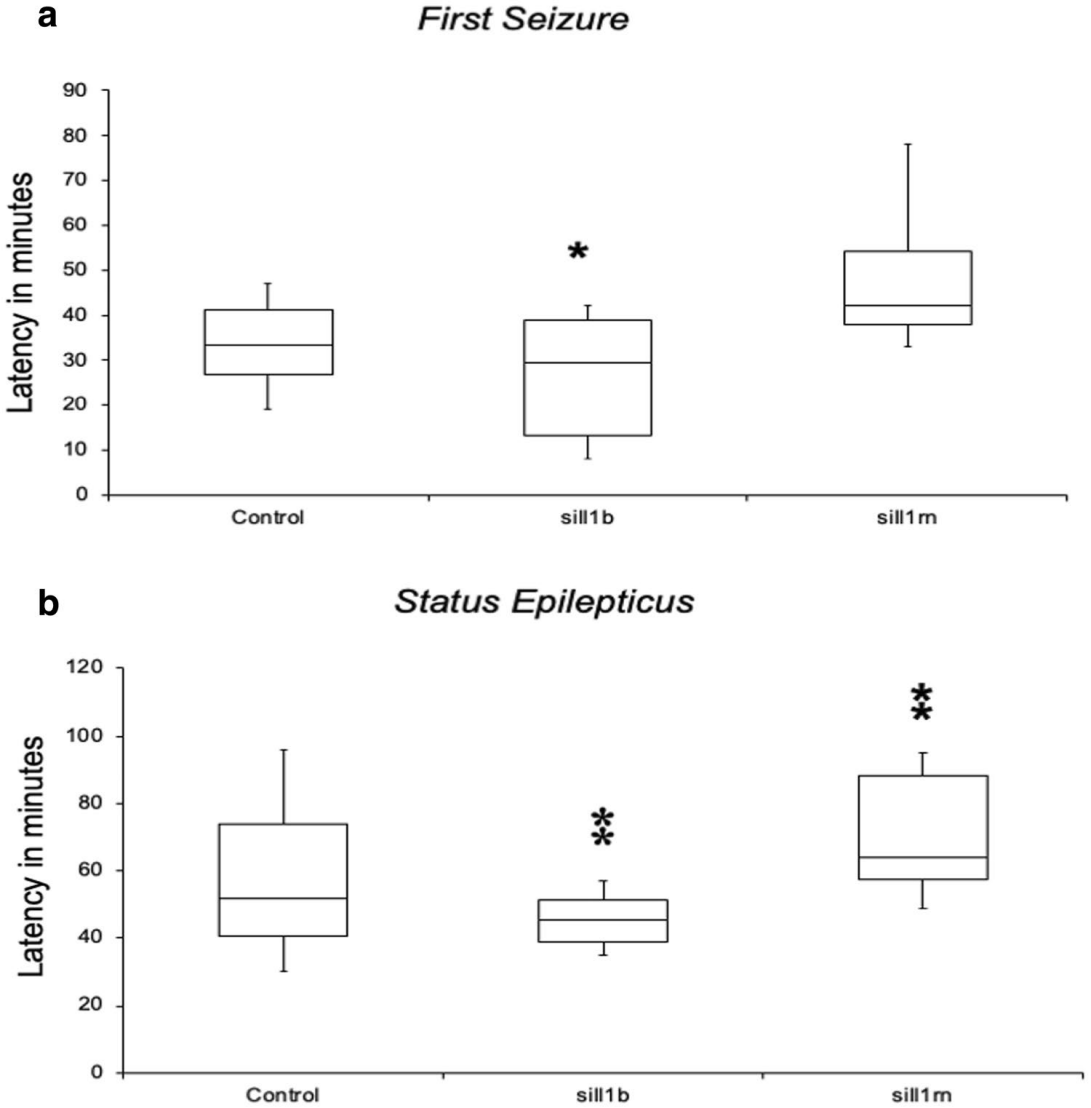
Phenotype assessment during the acute phase of the pilocarpine-induced epilepsy model. **a** The onset for the first seizure was 34 min (standard deviation [SD] = 12) in controls, 26 min (SD = 14) in animals pre-injected with *siIl1b*, and 49 min (SD = 16) in animals pre-injected with siIl1rn. **b** The onset of the *status epilepticus* (SE) was: 58 min (SD = 19) in controls, 46 min (SD = 25) in animals pre-injected with siIl1b, and 70 min (SD = 18) in animals pre-injected with siIl1rn. Single and double asterisk indicate a significant difference compared with the control group (analysis of variance with Tukey’s test, *p* < 0.05 and *p* < 0.01, respectively)

The time intervals between pilocarpine injection and the first observed seizure were: 34 min [standard deviation (SD) = 12] in controls, 26 min (SD = 14) in animals pre-injected with siIl1b (ANOVA and Tukey’s test, *p* < 0.05), and 49 min (SD = 16) in animals pre-injected with siIl1rn (Fig. 5A). There were no significant differences among the groups (ANOVA and Tukey’s test, *p* > 0.05).

In addition, there were significant differences in the onset of SE between the control group and both treated groups. The time intervals between pilocarpine injection and SE were: 58 min (SD = 19) in controls, 46 min (SD = 25) in animals pre-injected with siIl1b (ANOVA and Tukey’s test, p < 0.01), and 70 min (SD = 18) in animals pre-injected with siIl1rn (ANOVA and Tukey’s test, *p* < 0.01) (Fig. 5B).

### Slc1a3, a Glutamate Re-Uptake Transporter, was Downregulated 48 h After Il1b siRNA Injection

**Fig. 6:**
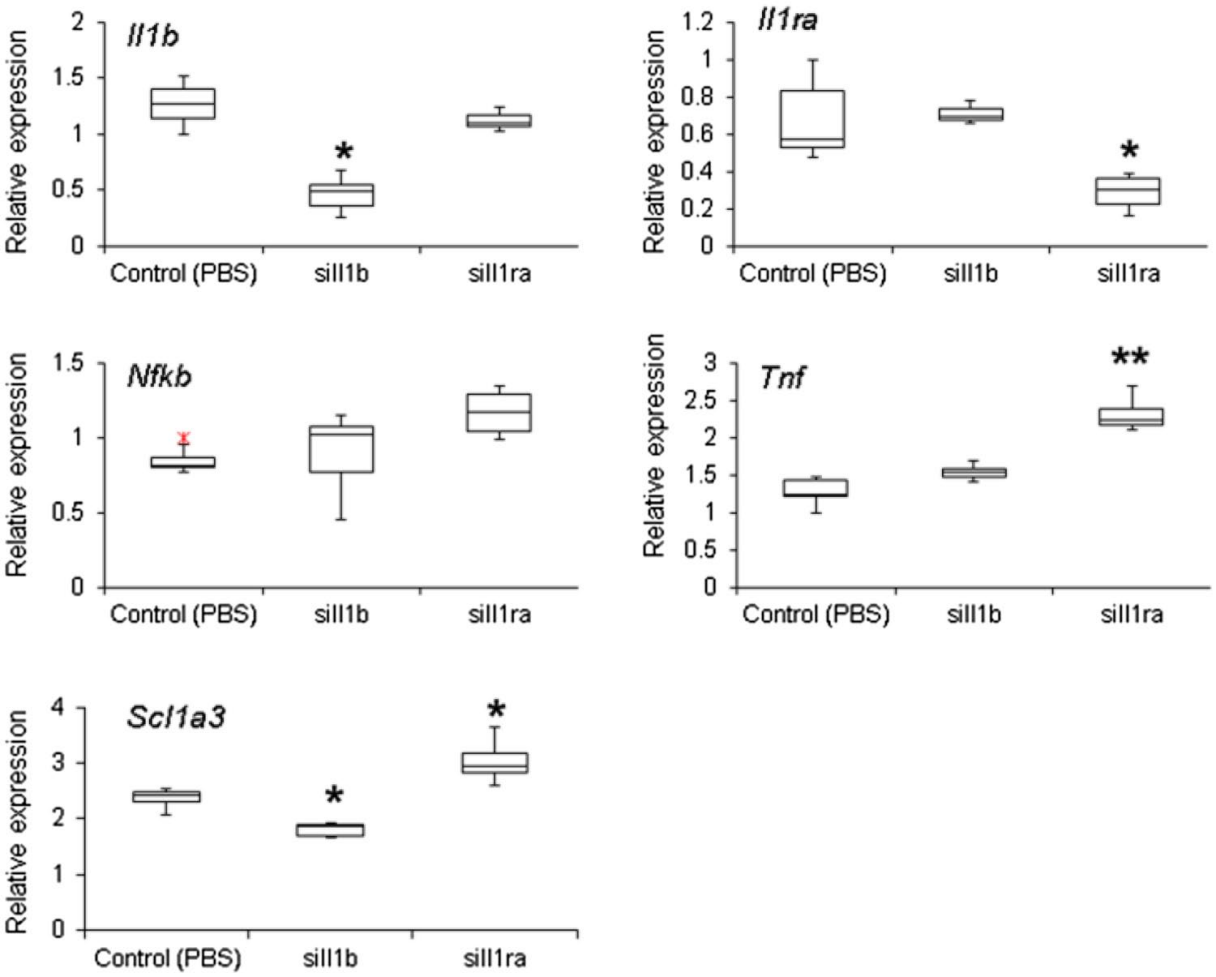
Analysis of gene expression 48 h after siIl1b or siIl1rn injection (no pilocarpine administration). Real-time polymerase chain reaction (qPCR) was used to determine the messenger RNA (mRNA) levels of *Il1b*, *Il1rn*, *Nfkb*, and *Slc1a3* in animals injected with *siIl1b* or *siIl1rn*. Control animals were injected with phosphate-buffered saline (PBS). All groups were composed of five animals. Single and double asterisk indicate a significant difference in gene expression (Kruskal–Wallis test, *p* < 0.05 and *p* < 0.01, respectively)

We analyzed the expression of several genes related to the Il1b signaling pathway as well as glutamate re-uptake in the synaptic cleft. We aimed to gain additional information regarding Il1b signaling pathway regulation, as well as to investigate the possible involvement of glutamate-mediated damage induced by changes in *Il1b* expression. Experiments were carried out 48 h after siRNA was injected so that the molecular effects of gene silencing were at the maximum. For genes related to the Il1b pathway, we observed an increase in *Tnf* expression after siIl1rn treatment. Furthermore, we observed a decrease in the mRNA expression of *Slc1a3* (also known as *Eaat1*), which is one of the most important glutamate re-uptake transporters in the CNS, when *Il1b* had been silenced (Fig. 6). The opposite effect (increased *Slc1a3* mRNA expression) was observed when *Il1rn* had been silenced.

### Il1rn Knockdown Induced an Increase in the Expression of Genes Known to be Overexpressed in the Silent Phase of the PILO Model

We investigated whether changes in the expression of several genes related to the silent phase of the PILO model could be induced by the transient knockdown of *Il1rn* in the acute phase. We found that genes expressed in the silent phase, such as *Npy*, *Plat*, and *Ntrk2* (Becker et al. 2003; He et al. 2004; Koyama and Ikegaya 2005), were upregulated in animals in which *Il1rn* had been silenced (Supporting information, Fig. S3). In addition, we still observed the upregulation of *Nfkb* induced by siIl1rn, which had been documented in the acute phase. Unfortunately, we could not perform a similar experiment in animals in which *Il1b* had been silenced due to the high mortality rate observed in that group.

### Transient Knockdown of Il1rn Decreased Neuronal Loss in the Hippocampus

**Fig. 7:**
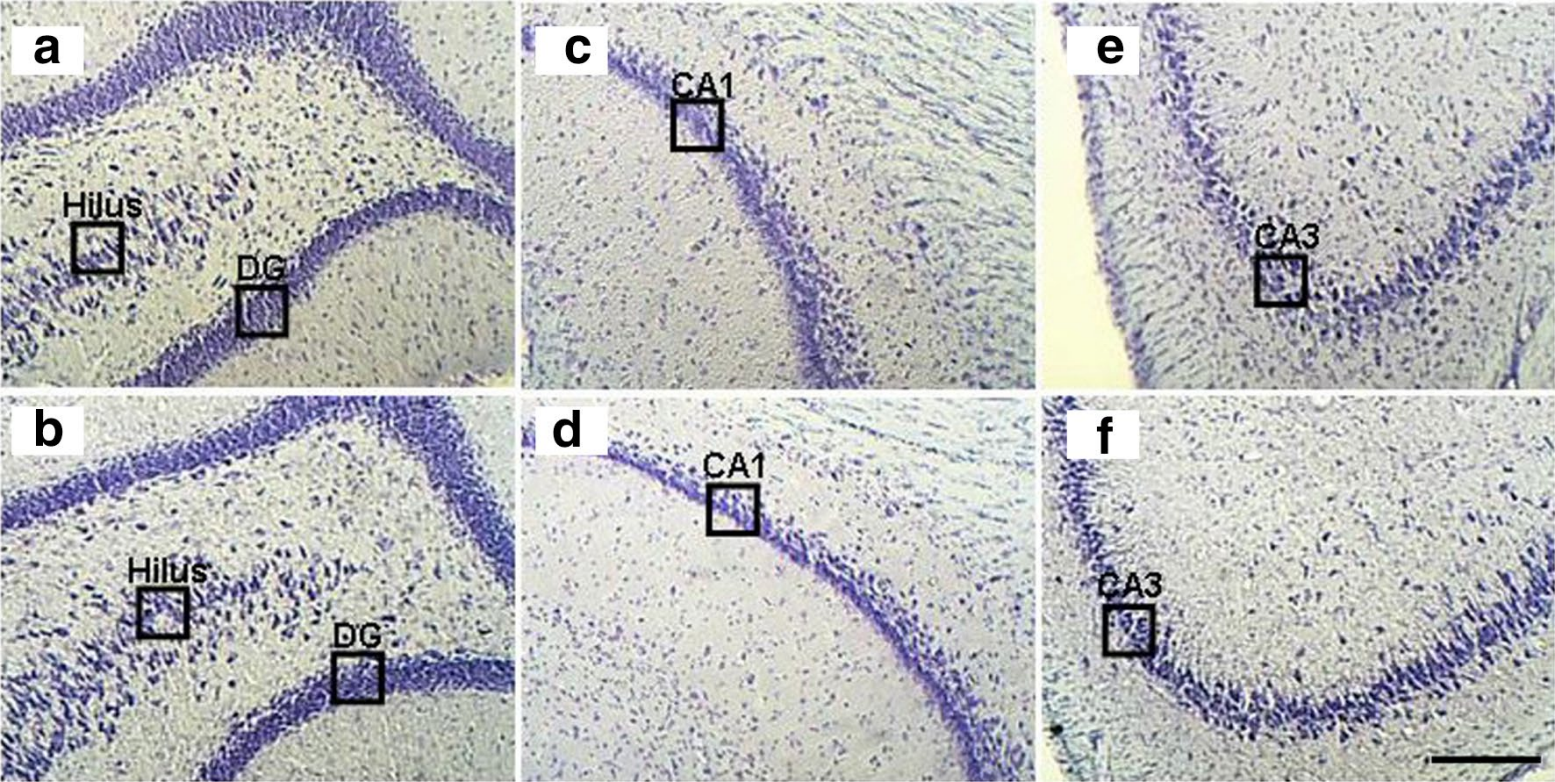
Nissl staining of the hippocampal subfields for neuronal count (cells/µm^2^) of animals in the chronic phase of the pilocarpine model. Animals injected twice (8-h interval between injections) with 25 μg of siIl1rn in the tail vein 48 h before pilocarpine administration and survived after *status epilepticus* were kept under observation for 90 days. The images show the following: **a** hilus and DG, **c** CA1, and **e** CA3 regions of control animals injected with pilocarpine and PBS; **b** hilus and DG, **d** CA1, and **f** CA3 regions of animals pre-treated with of siIl1rn. We observed a discrete neuronal loss in the hilus, DG, CA1, and CA3 regions in animals pre-treated with siIl1rn (ANOVA and Tukey’s test, *p* < 0.01). Scale bar 180 µm

To determine whether the molecular changes induced by the transient knockdown of *Il1rn* in the acute phase could lead to significant repercussions in the chronic phase of the PILO model, we analyzed hippocampal tissue 3 months after SE using histological staining (Fig. 7). Nissl staining revealed significant less neuronal loss in all the hippocampi fields of the animals in which *Il1rn* had been silenced (ANOVA and Tukey’s test, *p* < 0.01). The mean number of remaining, live cells in control versus *Il1rn* silenced groups are as follows, DG: 19.1 ± 0.6 *versus* 23.2 ± 0.3 cells/µm^2^; hilus: 7.4 ± 0.2 *versus* 8.4 ± 0.2 cells/µm^2^; CA1: 14.2 ± 0.4 *versus* 15.6 ± 0.4 cells/µm^2^ and CA3: 5.8 ± 0.2 *versus* 7.4 ± 0.3 cells/µm^2^.

## Discussion

In the present study, we explored the role of *Il1b* and its endogenous antagonist (*Il1rn*) in a rat model of MTLE induced by pilocarpine injection. For this endeavor, we used targeted delivery of siRNA by conjugating them to the RVG-9R peptide (Allan et al. 2005; Kim et al. 2010). We succeed in specifically downregulating *Il1b* and *Il1rn* for 72 h (Figs. 1, 2) without damaging the BBB (Fig. 3). We injected siRNA against *Il1b* and *Il1rn* 48 h before pilocarpine treatment due to the rapid increase in *Il1b*, but not *Il1rn*, observed 1 h after pilocarpine injection (Fig. 4). We found that knocking down endogenous *Il1b* in the CNS led to a decrease in the onset of the first seizure as well as SE (Fig. 5). By contrast, animals in which *Il1rn* has been silenced, and therefore presented increased Il1b signaling, showed the opposite effect (Fig. 5). Moreover, animals in which *Il1b* had been silenced showed an increase in mortality after 5 days of SE; the opposite effect was observed in animals in which *Il1rn* had been silenced (Supplementary information, Table S1). Even though we did not quantify *Il1b* and *Il1rn* mRNA transcripts or other inflammatory mediators such as interleukin-converting enzyme (ICE/caspase-1) during the acute phase of the PILO model, the data of the siRNA specificity (Fig. 6) and dynamics of *Il1b* and *Il1rn* expression after pilocarpine administration (Fig. 4) indicates that the effect observed during the acute phase of the PILO model in the siIl1b group is likely influenced by the specific knockdown of the *Il1b* gene and not by changes in expression of *Il1rn*, because the latter is not affected by *Il1b* silencing (Fig. 6). We also hypothesize that even with the activation of inflammatory cascades and specially ICE/caspase-1 during the acute phase of the PILO model, the lower expression of *Il1b* RNA in the siIl1b group ultimately produces less mature Il1b (Fig. 5). In addition, we observed that it takes at least 18 h after pilocarpine injection for *Il1rn* expression to increase in the brain (Fig. 4). This delay is surprising, and it contrasts with what has been observed in the periphery, where *Il1rn* shows an increased expression 4 h after an inflammatory insult (Zahedi et al. 1991), suggesting that the brain is much less effective than the periphery in inducing a crucial mechanism for rapidly terminating the effects of a sustained increase in endogenous Il1b.

Thus, the major finding of this study, as shown by the mortality observed after SE, and by the decrease in the expression of the glutamate reuptake transporter *Slc1a3* 48 h after *Il1b* knockdown, is that endogenous Il1b seems to influence animal survival after SE during the acute phase of the PILO model. Our interpretation for this observation is that by silencing *Il1b*, there is a decrease in the expression of *SCL1a3*; on the contrary, silencing the *Il1b* antagonist, using siRNA against *Il1rn*, increases the expression of *Scl1a3*. In this way, when Il1b is reduced there is a slower withdrawal of glutamate from the synaptic cleft, increasing its excitotoxic effect. Thus, decreasing the basal production of Il1b before SE somehow affects the CNS milieu and makes the tissue more vulnerable to hyperexcitability.

One hypothesis to explain the effect of Il1b in neuronal excitability is that it directly inhibits K^+^ efflux and increases Ca^2+^ influx and NMDA-R function, all of which contribute to seizure susceptibility and seizure-related damage (Meini et al. 2000; Viviani et al. 2003; Zhu et al. 2006; Eyo et al. 2014, 2018; Tian et al. 2017). More recently, several studies have begun to unravel the non-immunological roles played by endogenously produced Il1b in the brain; this molecule is crucial to maintain homeostasis and is far from neurotoxic (for an extensive review, see Hewett et al. 2012; Liu and Quan 2018). Furthermore, studies involving *IL1B* polymorphisms in humans have shown an association between a variant located at the promoter region of *IL1B* (C-511T), which produces less protein, and increased risk for epilepsy and febrile seizure (Wen et al. 2006; Al Morshedy et al. 2017). These findings support the hypothesis that endogenous Il1b may also have protective roles in the CNS and that insufficient endogenous Il1b predisposes the CNS to hyperexcitability. In addition, under physiological conditions, Il1b seems to modulate several functions in the CNS, such as regulating sleep (De Sarro et al. 1997; Krueger et al. 1998) and promoting learning and long-term potentiation (LTP) (Bellinger et al. 1993; Schneider et al. 1998; Ikegaya et al. 2003; del Rey et al. 2013). Chronic overexpression of *Il1b* in brain areas such as the hippocampus have been implicated in impaired spatial memory (Moore et al. 2009) and in contextual fear memory (Matousek et al. 2010) in conditional transgenic mice, and with seizure susceptibility in animal models of sleep fragmentation (Grubac et al. 2021) and prostatitis (Sutulovic et al. 2019). By contrast, transgenic mice with deficient Il1b signaling or pharmacological inhibition of CNS Il1b also present with memory and learning impairment (Avital et al. 2003; Goshen et al. 2007). More recently, it has been observed that NMDA-R together with Il1b appear to be necessary to induce the CX3CR1-dependent microglial process convergence (MPC)—an increase in the interaction between microglia and neurons, especially in the dendrites and axons, that confers neuroprotective effects in chemically induced animal models of epilepsy, thereby decreasing seizure severity and cell death (Eyo et al. 2016, 2018). Interestingly, the results described above seem to be in contradiction with the previously observed effect of Il1b-driven NMDA-R activation contributing to seizure severity (Meini et al. 2000; Viviani et al. 2003; Zhu et al. 2006; Eyo et al. 2014, 2018; Tian et al. 2017). However, these complex regulatory functions of Il1b are most likely time and dosage-dependent (Liu and Quan 2018), and any disruption in the balance of endogenous production may potentially lead to impaired functions.

Another important issue that is relevant for the interpretation of previous results in the literature and our current study is that there are relevant limitations to many previous studies regarding the strategies used to assess animal Il1b expression and activity or to deliver this molecule and its antagonists into the CNS. In most studies, researchers have used stereotactic surgery for this purpose (Panegyres and Hughes 1998; Vezzani et al. 1999, 2000, 2002; Yuhas et al. 1999; De Simoni et al. 2000; Yi et al. 2004; Dube et al. 2005; Heida and Pittman 2005; Sayyah et al. 2005). This procedure can potentially induce further inflammatory changes, which may make it challenging to study the genes involved in inflammatory pathways. Indeed, in the present study, we observed the activation of genes related to inflammation in the CNS after sham stereotactic surgery (Supporting information, Fig. S4). As pointed out by Hewett et al. (2012), the route of administration of the convulsant (local or systemic) when combined with exogenously applied Il1b can lead to contradictory roles for Il1b in the context of epileptogenesis.

Although researchers have reported remarkable results using siRNA in various organs, the CNS remains a difficult target mainly due to the protection of the BBB and to the degree of cellular diversity (Hassani et al. 2005; Pardridge 2007). Many protocols for the in vivo delivery of siRNA into the CNS use intrathecal injection associated with a cofactor, and stereotaxic surgery is needed in all cases (for a review, see Schlachetzki et al. 2004). In addition to their invasiveness, these approaches promote only restricted silencing, limited to the injection site. Furthermore, adenovirus and retrovirus transduction protocols promote gene silencing in a variety of cell types in the CNS; however, this is also a limited strategy because an injection into the cerebral parenchyma is needed.

Furthermore, inflammatory responses (triggered by the viral vector) may interfere with molecular and phenotypic analyses (Jooss and Chirmule 2003; Lowenstein and Castro 2003). Thus, the strategy of using siRNA conjugated to a short peptide derived from the RVG has emerged as a simple and less invasive strategy for promoting gene knockdown in different areas of the CNS; this method allows for the intravenous delivery of siRNA into the brain (Kumar et al. 2007). In our experiment, we observed effective gene knockdown in several areas restricted to the CNS (Supporting information, Fig. S2), and this information may be useful for planning future experiments. Even though this non-invasive in vivo delivery technique was effective for knocking down genes in the CNS, a lingering concern was the possibility of significant damage to the BBB by the use of siRNA::RVG-9R complexes. We were able to rule out this possibility using MRI, showing that treatment with different siRNA::RVG-9R complexes did not alter BBB permeability to molecules equal in size or larger than Gd-DTPA, the paramagnetic contrast used in our experiments (molecular weight of approximately 550 g/mol).

siRNA emerged in the early 2000s as a promising biological and therapeutic tool, but there are considerable limitations that need to be addressed when applying this method to functional studies (Jackson and Linsley 2010). Among these, off-target effects are of special concern (Jackson and Linsley 2004; Jackson et al. 2006). We assessed this issue carefully and only observed evidence of off-target effects when using high concentrations of siRNA (two 50-μg injections); in this scenario, we found that expression of *Ptgs2* was affected as described previously as evidence of the oversaturation of RNAi machinery (Tschuch et al. 2008). The gene silencing effects observed in this study were temporary and present only during the acute phase; nonetheless, we were able to detect molecular and phenotypic changes that persisted beyond this period in the group in which *Il1rn* has been knocked down. Indeed, we observed that animals in which *Il1rn* had been knocked down 48 h before pilocarpine injection displayed an even greater upregulation of genes known to be overexpressed in the silent phase of the PILO model (*Plat*, *Ntrk2*, and *Npy*; Fig. 6).

Surprisingly, the changes in gene expression induced by temporarily knocking down *Il1rn* 48 h before pilocarpine injection were able to induce a decrease in neuronal death, detected by Nissl staining, in the chronic phase of the PILO model, as indicated by the cell counting data. Previous experiments using the pre-treatment of hippocampal cell cultures with agents that activate nuclear factor kappa-light-chain-enhancer of activated B cells (NF-κB) have shown that this approach can prevent neuronal apoptosis in vitro, or that inhibition of NF-κB function results in apoptosis of rat PC12 cells (Mattson et al. 1997; Taglialatela et al. 1997). Thus, we hypothesize that modifying gene expression in the acute phase of the PILO model may have lasting effects.

## Conclusion

Even though our study has limitations, such as the absence of *Il1b* and *Il1rn* transcript quantification during the acute phase of the PILO model, we have provided suggestive evidence that endogenously produced *Il1b* seems to have a role in homeostatic maintenance in the CNS under physiological conditions. In addition, this effect appears to be relevant for animal recovery after the initial insult due to SE. Although pending further investigation, we believe that our results are sufficient to challenge the previous hypothesis that nonspecific inhibition of inflammation is universally beneficial and urge caution regarding the proposal of using broad-spectrum anti-inflammatory agents to prevent epilepsy after an insult (Vezzani et al. 2011). Furthermore, we have shown for the first time the feasibility of studying and manipulating neuroinflammation in the CNS in a noninvasive and specific way within the context of epilepsy. With the recent Food and Drug Administration (FDA) approval of drug therapy for hereditary transthyretin-mediated amyloidosis based on siRNA (FDA 2018), our findings open new perspectives for applying RNAi to diseases of the CNS and encourage the development of new noninvasive delivery methods targeting specific cell types.

## Supplementary Information

Below is the link to the electronic supplementary material.

Supplementary file1 (DOCX 3555 kb)

## Data Availability

Not applicable.
