# Supplementary material for: Modulating Expression of Endogenous Interleukin 1 Beta in the Acute Phase of the Pilocarpine Model of Epilepsy May Change Animal Survival
Source: Cell Mol Neurobiol. 2022 Jan 21;43(1):367–80. doi: 10.1007/s10571-022-01190-y (PMC9813089; doi:10.1007/s10571-022-01190-y)
Supplement: Supplementary file 1 — Supplementary file1 (DOCX 3555 kb) [file 10571_2022_1190_MOESM1_ESM.docx]

SUPPORTING INFORMATION

*online*

Modulating expression of endogenous interleukin 1 beta in the acute phase of the pilocarpine model of epilepsy may change animal survival

Pascoal V.D.B.^1,2‡^, Marchesini R.B.^1‡^, Athié M.C.P.^1^, Matos A.H.B.^1^, Conte F.F.^1^, Pereira T.C.^1,3^, Secolin R.^1,2^, Gilioli R.^4^, Malheiros J.M.^5,6^, Polli R.S.^7^, Tannús A.^5^, Covolan L.^6^, Pascoal L.B.^8^,Vieira A.S.^9^, Cavalheiro E.A.^10^, Cendes, F.^11^, & Lopes-Cendes I.^1^

1 – Department of Medical Genetics and Genomic Medicine, School of Medical Sciences, University of Campinas (UNICAMP), and the Brazilian Institute of Neuroscience and Neurotechnology (BRAINN), Campinas, SP, Brazil

2 – Department of Basic Science, Fluminense Federal University, Nova Friburgo, Rio de Janeiro, Brazil

3 – Department of Biology, Faculty of Philosophy, Sciences and Letters at Ribeirao Preto, University of Sao Paulo (USP), Ribeirao Preto, SP, Brazil

4 – Multidisciplinary Centre for Biological Investigation (CEMIB), University of Campinas (UNICAMP), Campinas, SP, Brazil

5 – Centro de Imagens e Espectroscopia por Ressonancia Magnetica (CIERMag), Institute of Physics, University of Sao Paulo (USP), Sao Carlos, SP, Brazil

6 – Department of Physiology, Federal University of Sao Paulo (UNIFESP), Sao Paulo, SP, Brazil

7 – Institute of Science and Technology, Federal University of São Paulo, São José dos Campos, SP, Brazil

8 – Laboratory of Cell Signaling, School of Medical Sciences, University of Campinas – (UNICAMP), Campinas, SP, Brazil

9 – Department of Structural and Functional Biology, Institute of Biology, University of Campinas – (UNICAMP), Campinas, SP, Brazil

10 – Department of Neurology and Neurosurgery, Federal University of Sao Paulo, (UNIFESP), Sao Paulo, SP, Brazil

11 – Department of Neurology, School of Medical Sciences, University of Campinas –(UNICAMP); and the Brazilian Institute of Neuroscience and Neurotechnology (BRAINN), Campinas, SP, Brazil

**Supporting information Table S1** Differences in animal mortality per group observed during pilocarpine treatment (day 1) and over the following 5 days (days 2–5)

|  | **Control** | **siIl1b** | **siIl1rn** |
| --- | --- | --- | --- |
| **Number of deaths (day 1)** | 8 (42%) | 4 (44%) | 2 (18%) |
| **Number of deaths (days 2–5)** | 1 (5%) | 2 (22%) | 1 (9%) |
| **Total deaths (days 1–5)** | 9/19 (47%) | 6/9 (66%)* | 3/11 (27%)* |

*chi-squared test, p < 0.05 compared with the control group

#### FIGURES


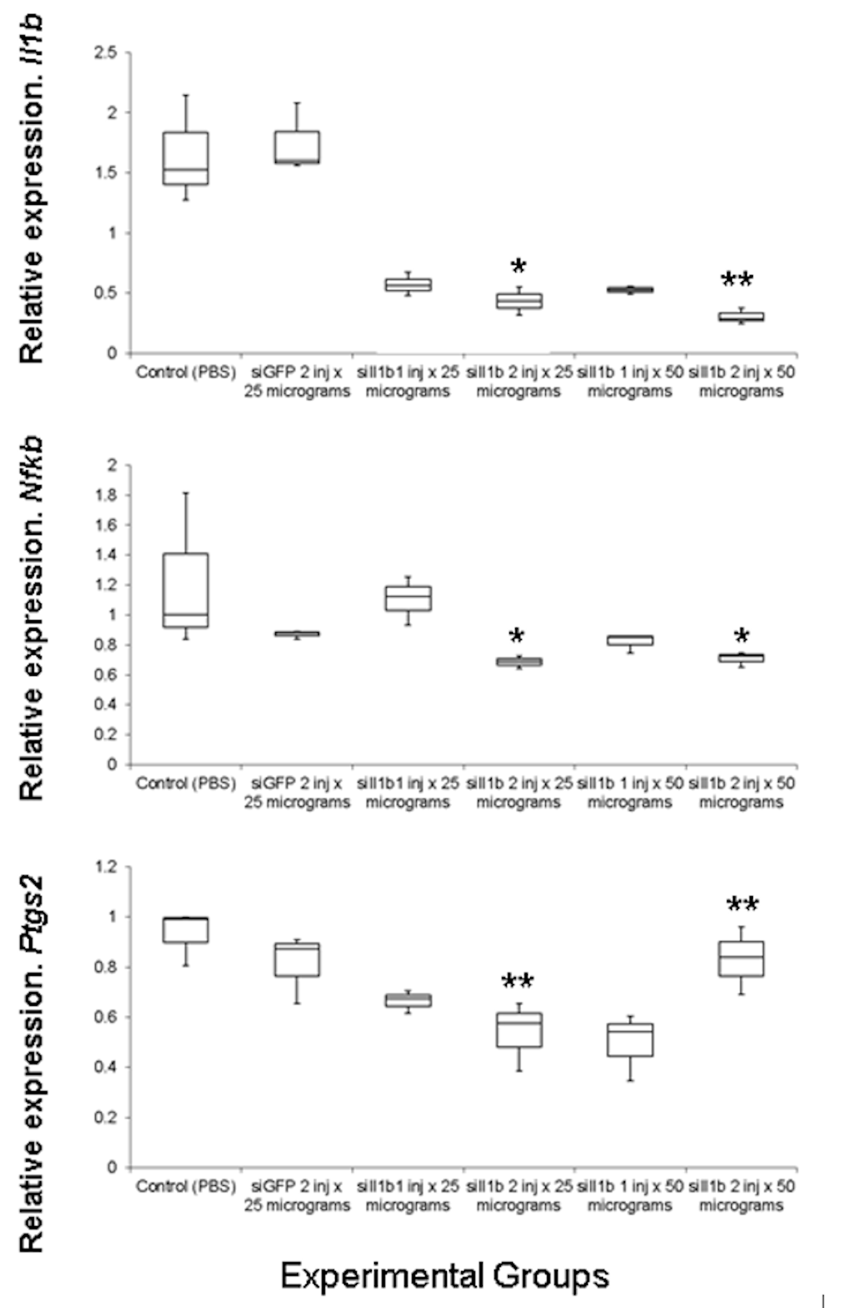


**Supporting information Figure S1.** Dose-response curve of gene silencing effects after siIl1b intravenous tail injection in rats. The efficiency of gene silencing in whole brain was enhanced with an increased number of injections and increased mass of small interfering RNA (siRNA). We investigated the expression of the target gene *Il1b*, as well as of two biologically related genes, *Ptgs2* and *Nfkb*. All groups were composed of five animals. * and ** indicate significant silencing in the group injected twice with siIl1b (8-h interval between injections) using 25 or 50 µg (Kruskal–Wallis test, p < 0.05 and p < 0.01, respectively)


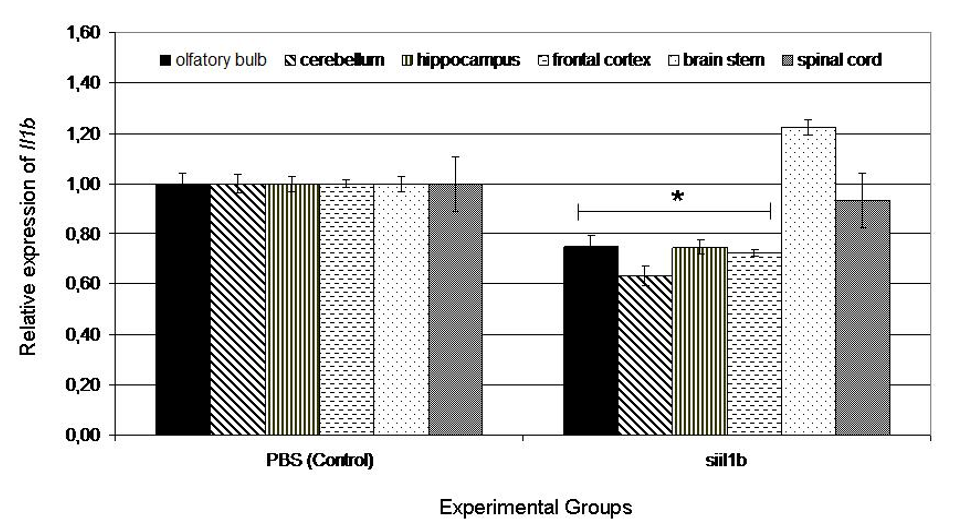


**Supporting information Figure S2** Gene silencing effects in different brain regions. Animals were injected twice (8-h interval between injections) with 25 μg of siIl1b in the tail vein. Gene expression was quantified by real-time polymerase chain reaction (qPCR) 48 h post-injection. Gene silencing was observed in four out of five brain regions: the olfactory bulb, the cerebellum, the hippocampus, and the frontal cortex. Control animals were injected with phosphate-buffered saline (PBS). All groups were composed of five animals. * indicates significant gene silencing (Mann–Whitney test, p < 0.05). The vertical bars indicate the standard deviation


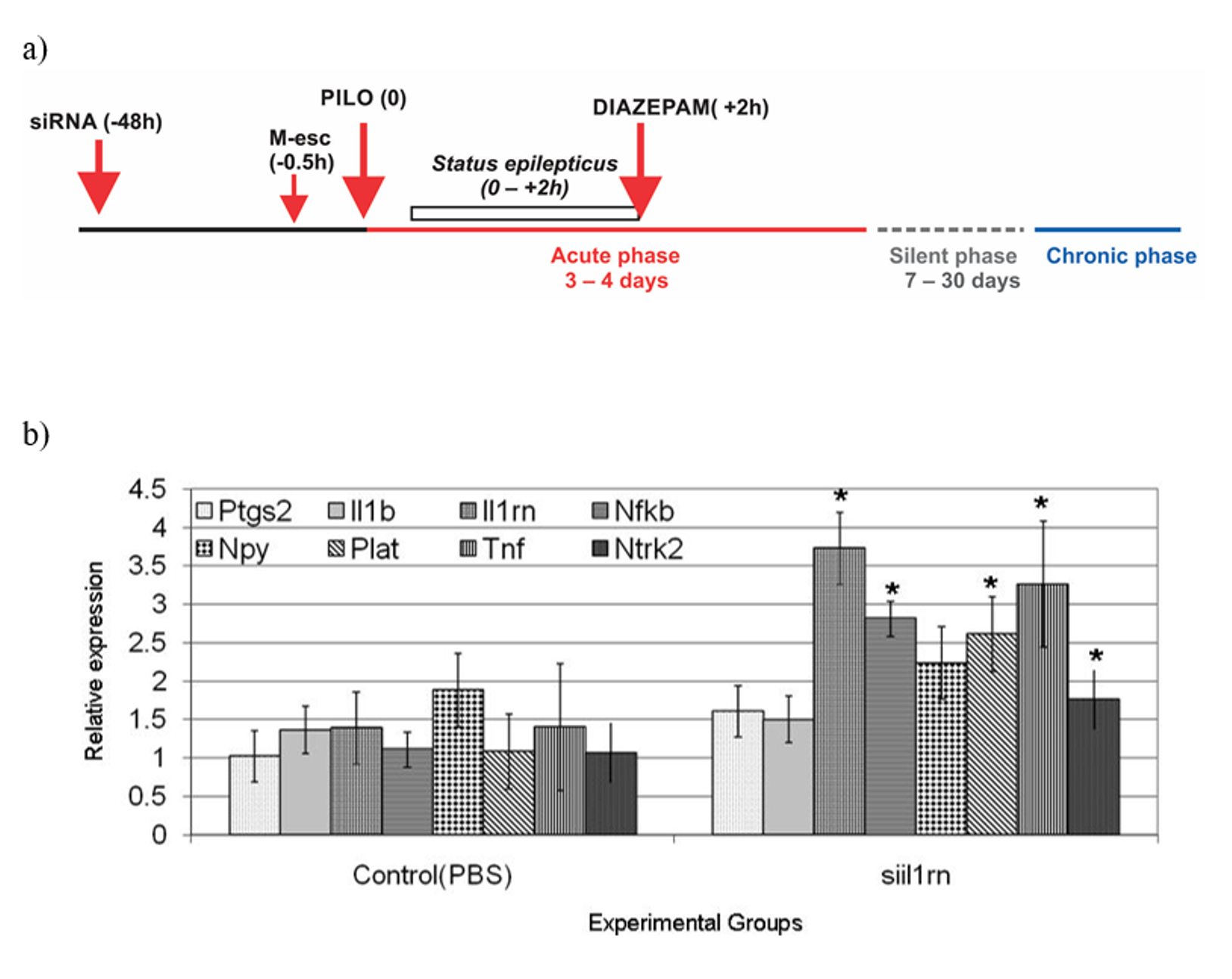


**Supporting information Figure S3** Relative quantification of eight genes in animals pre-treated with siIl1rn and injected with pilocarpine. (a) Animals were injected twice (8-h interval between injections) with 25 μg of siIl1b in the tail vein 48 h before pilocarpine administration. (b) The messenger RNA (mRNA) levels of eight genes were determined in the brain five days after *status epilepticus* (SE). We observed a significant increase in *Il1rn*, *Tnf*, *Nfkb*, *Plat*, and *Ntrk2* mRNA levels in animals pre-treated with *siIl1rn* compared with the control group (treated with phosphate-buffered saline [PBS]). By contrast, there was no change in the expression of *Npy*, *Ptgs2*, and *Il1b*. All groups comprised five animals. * indicates a significant difference in expression (analysis of variance and the Tukey test, p < 0.05). Vertical bars indicate the standard error


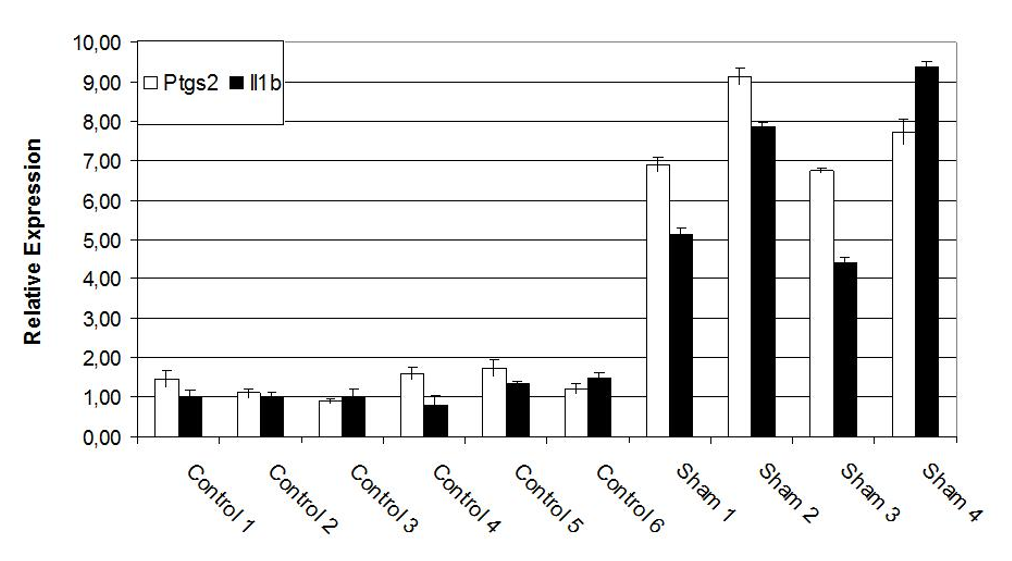


**Supporting information Figure S4** Relative quantification of *Ptgs2* and *Il1b* genes related to inflammation in the central nervous system after sham stereotactic surgery. The *Ptgs2* and *Il1b* messenger RNA (mRNA) levels were determined in the hippocampus of control animals and animals 5 days after stereotactic surgery. We observed a significant increase in *Il1b* and *Ptgs2* mRNA levels in animals in the sham group compared with the control group (analysis of variance and Tukey post-hoc test, p < 0.05). Vertical bars indicate the standard deviation
